# Do Large Carnivores and Mesocarnivores Have Redundant Impacts on Intertidal Prey?

**DOI:** 10.1371/journal.pone.0170255

**Published:** 2017-01-13

**Authors:** Justin P. Suraci, Michael Clinchy, Liana Y. Zanette

**Affiliations:** 1 Department of Biology, Western University, London, ON, Canada; 2 Raincoast Conservation Foundation, Sidney, BC, Canada; University of Sydney, AUSTRALIA

## Abstract

The presence of large carnivores can affect lower trophic levels by suppressing mesocarnivores and reducing their impacts on prey. The mesopredator release hypothesis therefore predicts prey abundance will be higher where large carnivores are present, but this prediction assumes limited dietary overlap between large and mesocarnivores. Where dietary overlap is high, e.g., among omnivorous carnivore species, or where prey are relatively easily accessible, the potential exists for large and mesocarnivores to have redundant impacts on prey, though this possibility has not been explored. The intertidal community represents a potentially important but poorly studied resource for coastal carnivore populations, and one for which dietary overlap between carnivores may be high. To evaluate usage of the intertidal community by coastal carnivores and the potential for redundancy between large and mesocarnivores, we surveyed (i) intertidal prey abundance (crabs and fish) and (ii) the abundance and activity of large carnivores (predominantly black bears) and mesocarnivores (raccoons and mink) in an area with an intact carnivore community in coastal British Columbia, Canada. Overall carnivore activity was strongly related to intertidal prey availability. Notably, this relationship was not contingent on carnivore species identity, suggestive of redundancy–high intertidal prey availability was associated with either greater large carnivore activity or greater mesocarnivore activity. We then compared intertidal prey abundances in this intact system, in which bears dominate, with those in a nearby system where bears and other large carnivores have been extirpated, and raccoons are the primary intertidal predator. We found significant similarities in intertidal species abundances, providing additional evidence for redundancy between large (bear) and mesocarnivore (raccoon) impacts on intertidal prey. Taken together, our results indicate that intertidal prey shape habitat use and competition among coastal carnivores, and raise the interesting possibility of redundancy between mesocarnivores and large carnivores in their role as intertidal top predators.

## Introduction

The structure of mammalian carnivore communities (i.e., the relative abundance of large carnivores and mesocarnivores) can be a major determinant of species abundance across food webs. Because large carnivores suppress mesocarnivore foraging, the community of species that mesocarnivores eat may differ substantially depending on the presence or absence of large carnivores [1–5]. Thus, the mesopredator release hypothesis [6,7] predicts that prey abundance and diversity will be higher where large carnivore abundance is high, relative to where large carnivores have been reduced or extirpated. This prediction will hold when dietary overlap between large carnivores and mesocarnivores is limited, for instance, when considering large carnivore populations (e.g., many continental populations of wolves *Canis lupus* and cougars *Puma concolor*) that depend primarily on large ungulate prey [8–10], and thus exhibit narrower diets than sympatric mesocarnivores, which tend to be more omnivorous [11,12].

The consequences for the prey community are less clear when both large carnivores and mesocarnivores compete for the same resources. Intraguild predation theory suggests that predator species subject to suppression by and competition with a dominant predator may be more efficient at exploiting a shared resource [13–15], and thus the presence of large carnivores may benefit shared prey by suppressing the foraging of more efficient mesocarnivores. Alternatively, different carnivore species may be largely redundant in their impacts on prey, in which case differences in the carnivore community would be expected to have only minimal effects on prey abundance. Functional redundancy occurs when adding one or more species to a functional group does not substantially affect that group’s role in the community [16–19], and has been considered in assessing the impacts of multiple large carnivore species on shared ungulate prey [20,21]. However, the potential for redundancy has not been considered in studies of mammalian mesopredator release, which, as noted above, typically focus on large carnivore and mesocarnivore species with limited dietary overlap [3,6,7,11], and thus often detect substantial differences in the prey community in the presence and absence of large carnivores (e.g., [3,4]). Yet when large carnivores and mesocarnivores utilize the same resources, little change in the shared prey community may be expected if the potential exists for released mesocarnivores to assume the functional role of an extirpated large carnivore, e.g., through increased abundance and/or per capita impacts on prey [22–24], as has been demonstrated among invertebrate predators [19]. This may be particularly likely when the carnivore community contains highly omnivorous large carnivores and mesocarnivores with substantial dietary overlap [25,26]

For populations living at the terrestrial-marine interface, the potential for dietary overlap between carnivore species may be high. Marine derived foods, ranging from spawning fish to marine mammal carcasses, provide a major source of energy and nutrients to many coastal carnivore populations [18,27–29], a notable example being that of Pacific salmon (*Oncorhynchus* spp.), which are consumed by a large number of carnivore species across the Pacific coast of North America every year during annual salmon runs [30–33]. Salmon are a particularly important food source for large carnivores–wolves [9,29] and bears (*Ursus* spp.; [33–35])–which, due to dietary overlap, may compete strongly for salmon in some areas [29], and whose movement of salmon carcasses away from spawning streams has widespread ecosystem-level effects, spreading marine-derived nutrients throughout the terrestrial community [31,32]. The abundant invertebrates and small fish that are year-round residents in rocky intertidal and shallow subtidal habitats (hereafter referred to as “intertidal prey”) represent a similarly rich food source for coastal carnivore populations, and omnivorous foragers such as black bears (*Ursus americanus*) and raccoons (*Procyon lotor*), as well as largely aquatic foragers such as American mink (*Neovison vison*), overlap in their use of these intertidal prey [28,30,36–38]. However, unlike the well-described salmon-carnivore relationship, surprisingly few studies have gone beyond simply describing carnivore use of intertidal prey [28], providing a limited understanding of the ecological importance of this interaction, i.e., whether the availability of intertidal prey affects the terrestrial carnivore community and whether carnivore foraging in turn impacts intertidal prey.

In one of the only studies to consider the impacts of a mammalian carnivore on intertidal prey, Suraci et al. [5] experimentally demonstrated that carnivore foraging can indeed have widespread effects across the intertidal community, sharply reducing the abundance of the carnivore’s intertidal prey, and, through predation on other intertidal predators (e.g., crabs), cause further cascading effects on species not directly subject to carnivore predation [5,36]. Thus, mammalian carnivores can act as top predators in intertidal communities. This research was conducted on mesocarnivore raccoons in a system (the Gulf Islands of British Columbia, Canada) from which all native large carnivores were extirpated by humans a century ago [36], thereby releasing raccoons both from large carnivore predation, and from potential competition with large carnivores for access to intertidal resources. The mesopredator release hypothesis would predict that, where large carnivores persist, mesocarnivore impacts would be lessened and prey abundance would therefore be greater, but the relative abundance of intertidal prey in habitats with an intact carnivore community, i.e., one consisting of the full suite of large carnivores and mesocarnivores, remains unknown.

Here we examine the relationship between carnivores and intertidal prey in a coastal system supporting an intact carnivore community. Clayoquot Sound (hereafter “Clayoquot”), in British Columbia, Canada, supports three large carnivore species (black bears [hereafter, “bears”], wolves, and cougars), as well as several mesocarnivore species, including raccoons and mink [39]. With over 1,300 km of shoreline and many protected bays and inlets [40], there is ample opportunity for carnivores here to exploit intertidal prey. Bears in Clayoquot regularly forage in intertidal habitats [40], and have been observed to consume several of the same intertidal species (e.g., intertidal fish and shore crabs *Hemigrapsus* spp.; [40]) known to be important prey for mesocarnivores (raccoons and mink; [30,36]). Thus the potential exists for competition between large carnivores and mesocarnivores, and for redundancy in their impacts on intertidal prey (i.e., intertidal invertebrates and fish). In the present study we consider the interaction between the intertidal prey and coastal carnivore communities, showing that the availability of intertidal resources affects the distribution and community composition of carnivores that forage in the intertidal (hereafter, “intertidal carnivores”), but that the relative abundance of large carnivores and mesocarnivores at a site does not appear to affect intertidal prey, suggesting that redundancy in the impacts of different carnivore species may exist. We then compare intertidal prey abundances in Clayoquot with those in the nearby Gulf Islands, a system which lacks large carnivores [36], providing further evidence suggestive of redundancy between large carnivores and mesocarnivores in their impacts on intertidal prey.

## Methods

### Overview and study area

Clayoquot is located on the remote central west coast of Vancouver Island, BC, Canada (49°23′ to 49°04′ N, 126°13 to 125°45′). Terrestrial habitat in Clayoquot consists of old growth and second growth temperate rainforest dominated by coastal western hemlock (*Tsuga heterophylla*), and the adjacent shoreline is a complex of rocky intertidal and mudflat habitat. The present study covered approximately 156 km^2^ in the southeastern section of Clayoquot, occurring entirely within the Clayoquot Sound Biosphere Reserve, a UNESCO World Heritage Site.

Ten shoreline sites, separated by an average minimum distance of 3.1 (± 1.4 SD) km, were chosen based on the presence of cobble-boulder shoreline, an intertidal habitat type in which all of the large carnivore and mesocarnivore species noted above were observed during preliminary surveys, and which is known to be productive foraging habitat for many carnivore species, given the availability of intertidal invertebrates and fish [30,36,40]. All sites were located in sheltered waters several kilometers from the open ocean. At each site we estimated (1) the relative abundance of large carnivores and mesocarnivores using camera traps and scat surveys, and (2) the availability of marine prey using intertidal quadrat sampling and shallow subtidal crab trapping.

### Carnivore relative abundance

At each site, we deployed three camera traps (Moultrie M-990i, Moultrie Products, LLC, USA), spaced 154 (± 68 SD) m apart. Camera traps were set along the shoreline just above the high tide line (mean ± SD distance to forest edge = 1.1 ± 1.5 m) at an average height of 0.85 (± 0.26 SD) m, and directed towards the water to be triggered by mammals foraging or transiting in the intertidal. We confirmed that aspects of camera set-up did not differ between sites in ways that could affect the ability of camera traps to capture images of carnivores in the intertidal, using Spearman’s rank correlation to test whether site-level average values of four camera set-up properties were correlated with the number of carnivore images recorded per camera-day (minimum distance to a neighboring camera: *P* = 0.26, slope of shoreline on which the camera was set: *P* = 0.73, distance from the forest edge: *P* = 0.89, camera height: *P* = 0.56). Two camera traps at each site were programmed to record photographs (3 photos per triggering event) and the third camera was programmed to record 90 s (day) or 30 s (night) videos. All cameras were programmed to retrigger after a 5 s delay. Camera traps were set between 14 June and 27 August 2015. At each site, the mean number of days during which at least one camera was active was 73.3 (range 71 to 75) d, resulting in a total of 733 camera-days across all sites. When quantifying the relative abundance of mammalian carnivores from camera trap images and videos, we considered recordings of a given species as independent occurrences only if they were separated by at least 60 min from recordings of the same species on any of the three cameras at a site (i.e., within a site, camera traps were considered to be non-independent). Preliminary review of camera trap records indicated that this 60 min cut-off minimized the likelihood of recounting the same individual passing in front of the camera trap multiple times during a single intertidal foraging bout, and is relatively conservative compared to the cut-off used in previous camera trap studies of mammalian carnivores [5,41,42]. In all results reported below, we express the relative abundance of carnivores as individuals per camera-day, where camera-days are the total number of days that at least one camera was active at a site (Table 1).

**Table 1:** Relative abundance of large carnivores and mesocarnivores at the ten study sites, as determined by camera trapping and scat transects.

	Individuals camera-day^-1^							Scats 500 m^-1^				
Site	Black Bear	Wolf	Cougar	Raccoon	Mink	All Large Carnivore	All Meso-Carnivore	Black Bear	Wolf	Raccoon	Mink	All Meso-Carnivore
Bear Bay	0.95	0	0.01	0	0.03	0.96	0.03	14.0 (±6.81)	0	0	5.0 (±1.58)	5.0 (±1.58)
Fundy Creek	0.04	0	0.01	0.18	0.24	0.05	0.42	0	0	6.25 (±2.66)	13.25 (±4.82)	19.5 (±5.30)
Grice Bay	0	0.31	0	0.01	0.07	0.31	0.08	0	0.25 (±0.25)	0.25 (±0.25)	4.75 (±0.48)	5 (±0.41)
Gunner Inlet	0.39	0	0	0.01	0	0.39	0.01	6.25 (±2.02)	0	0	2.0 (±1.08)	2.0 (±1.08)
Indian Bay	0.34	0.01	0	0.15	0.01	0.35	0.16	4.25 (±1.25)	0	0.5 (±0.29)	4.25 (±1.38)	4.75 (±1.65)
North Fortune	0.57	0	0.01	0	0.03	0.58	0.03	13.25 (±4.33)	0	0	2.0 (±1.08)	2.0 (±1.08)
Orca Bay	1.37	0	0	0	0.03	1.37	0.03	10.0 (±2.12)	0	0	3.0 (±1.41)	3.0 (±1.41)
Southeast Meares	0	0	0	0.08	0.26	0	0.35	0	0	5.5 (±2.10)	16.25 (±2.32)	21.75 (±3.28)
Tofino Inlet	0.07	0	0	0.11	0.01	0.07	0.13	1.33 (±0.33)	0	1.0 (±0.58)	2.33 (±1.33)	3.33 (±1.86)
Tranquil Cove	0.33	0	0	0.03	0.03	0.33	0.06	3.25 (±1.11)	0	0.25 (±0.25)	1.0 (±0.41)	1.25 (±0.63)

Relative abundance estimates from scat transects are presented as the mean (± SE) of four 500-m transect sections walked at each site.

We conducted scat transects twice at each site between 01 July and 25 August 2015, with the two sampling occasions at a given site separated by at least 23 days (mean ± SD = 32 ± 8 d). Scat transects covered 1 km of shoreline, centered on the location of the camera traps, and were divided into two 500-m sections [43]. Two researchers conducted all scat transects, with each individual searching one 500-m section on foot during each sampling occasion. Transects covered both the high shore, extending ~ 5 m out from the forest edge, and the section of forest immediately adjacent to the shoreline, extending ~ 15 m in from the tree line. For all mammal scats encountered, the researcher noted the species, along with an estimate of their certainty regarding species identification (high, medium, or low). Only scats identified with high or medium certainty were included in the analyses described below. Carnivore relative abundance estimates from scat transects were expressed as the number of scats encountered per 500 m (Table 1).

### Marine prey availability

Previous work in the Gulf Islands, BC, Canada (located off the southeast coast of Vancouver Island, approximately 140 km from our Clayoquot study sites) indicated that a suite of intertidal and shallow subtidal species, which occur in both the Gulf Islands and Clayoquot, are common prey items for mammalian carnivores foraging in the intertidal [36], and observations in Clayoquot confirm that these prey items are also commonly used by bears in this region [40]. In estimating marine prey availability in Clayoqout, we therefore focused our sampling efforts on this suite of marine species, which includes shore crabs (*Hemigrapsus oregonensis* and *H*. *nudus*), prickleback fish (family Stichaeidae), and red rock crabs (*Cancer productus*). The abundance of shore crabs and prickleback fish was estimated using intertidal quadrat sampling, following methods described previously [5,36]. Briefly, at each site we sampled ten 0.25 x 0.25 m^2^ quadrats in both the high and mid intertidal zones (see [36] for a description of intertidal zones). Quadrats were spaced at 5-m intervals along a 50-m transect line that ran parallel to the water line, and all quadrat sampling was conducted within the section of shoreline covered by camera traps. Following Suraci et al. [36], all shore crabs were grouped into three size classes based on carapace width: small ≤ 1.3 cm, medium = 1.31–2.0 cm, large > 2.0 cm. Previous work indicated that carnivore predation on shore crabs is largely restricted to the two larger size classes [36], and we therefore focused our sampling efforts on medium and large shore crabs. Quadrat sampling took place between 03 June and 17 July 2015.

Following Suraci et al. [36], we estimated the abundance of red rock crabs across 1 km of shoreline at each site (centered on the location of the camera traps) by setting ten collapsible mesh crab traps in the shallow subtidal zone, just below the lowest low water line. Traps were spaced at 100-m intervals, baited with ~200 g of frozen herring, and left in place for 24 hrs. Red rock crabs undertake daily movements from the shallow subtidal into the intertidal [44] where they become available to terrestrial mammalian predators, and we previously reported that raccoon predation on red rock crabs in the intertidal affects the size of the shallow subtidal population [5,36]. The abundance of red rock crabs in the shallow subtidal thus provides a useful estimate of availability of red rock crabs to intertidal foraging carnivores. Crab trapping was performed between 02 June and 27 July 2015.

### Ethics statement

All sampling was conducted in accordance with of the Canadian Council on Animal Care and approved by the University of Victoria Animal Care and Use Committee (protocol numbers 2015-004(1) and 2014-021(1)). Marine prey sampling was approved by Fisheries and Oceans Canada (scientific license number XR 108 2015). Fieldwork was carried out on public lands, on the traditional territory of the Tla-o-qui-aht First Nation (with permission from the Tla-o-qui-aht Chief and Council), and within the Pacific Rim National Park Reserve (under permit number PRN-2015-18256).

### Statistical analysis

#### Large carnivore-mesocarnivore interactions

For all models described below, model assumptions were checked using statistical tests for normality and homogeneity of variance, and model fit was visually inspected using residual versus fitted value plots and quantile–quantile plots [45]. All analyses were conducted in R [46].

To investigate whether large carnivores may restrict mesocarnivore use of intertidal habitats in Clayoquot, we first tested for an effect of the relative abundance of large carnivores on mesocarnivore relative abundance using camera trap data (i.e., individuals per camera-day), treating sites (n = 10) as independent sampling units. We used generalized linear models (GLM) with a quasi-Poisson error distribution (to account for overdispersion in response variables [45]), using the total number of mesocarnivore recordings at a given site as our response variable, and including number of camera-days at the site as an offset to correct for sampling effort. The explanatory variable in all models was the number of large carnivores per camera-day. Four GLM analyses were performed with camera trap data. (1) We first analyzed the effect of all large carnivores (bears, wolves, and cougars) on all mesocarnivores (raccoons and mink) by pooling large carnivore and mesocarnivore recordings at a given site. As discussed below, bears were the numerically dominant large carnivore at most sites (indicated by both camera traps and scat transects) and are known to regularly exploit intertidal food [37,40]. Thus we also tested for an effect of bear relative abundance on the relative abundance of (2) all mesocarnivores, (3) just raccoons, and (4) just mink. The significance of the effect of large carnivore abundance in all quasi-Poisson GLM models was assessed using F-tests [45].

Large carnivores were detected at nine of the ten study sites and, as mentioned above, at most of these sites, bears were the numerically dominant large carnivore (see Table 1 and Results). The sole exception to this pattern was Grice Bay, one of only two sites at which bears were never detected. Grice Bay is the only site at which wolves were regularly recorded on camera traps (Table 1), with one or two individual wolves being recorded on 20 occasions (approximately every 4 days) throughout the duration of the study. Wolves are known to prey heavily on mesocarnivores in Clayoquot [47], and have been noted to harass and kill bears here and elsewhere [9,48–50]. Thus the absence of bears at Grice Bay may be attributable to the relatively high rates of wolf activity at this site, which may also affect habitat use by mesopredators. For this reason, the analyses of camera trap data that focused on interactions between bears and mesopredators (GLMs 2, 3, and 4 above) were run both with (n = 10 sites) and without (n = 9 sites) data from Grice Bay. Conclusions regarding the effects of bear relative abundance on mesopredator relative abundance were not affected by excluding Grice Bay (significance of bear abundance, as determined by F-tests, was unaffected). Nonetheless, given the potential confounding effect of high wolf activity at Grice Bay on both bear and mesocarnivore relative abundance at this site, we have chosen to present results below for models from which Grice Bay was excluded.

Scat transect data provides a complementary, but more coarse-scale estimate of carnivore relative abundance compared to that provided by camera traps, and was thus used solely to corroborate the general pattern of large carnivore and mesocarnivore relative abundance detected in camera trap data. Bear scats constituted all but one of the large carnivore scats detected (99.5% of 209 large carnivore scats; a single wolf scat was found at Grice Bay, Table 1), and preliminary investigation of scat data suggested that the number of mesocarnivore scats showed a threshold effect in relation to bear abundance, being relatively high at sites at which no bear scats were found, and then dropping off quickly and remaining low at all sites where bear scats were present. We therefore tested for an effect of bear presence or absence (based on the presence or absence of scats) on the relative abundance of mesocarnivore scats detected, categorizing sites at which no bear scats were found as “bear-absent” (n = 3) and sites at which at least one bear scat was found as “bear-present” (n = 7). Consistent with scat transects providing more coarse-scale data than camera traps, one of the study sites at which no bear scats were found (Fundy Creek) showed low but detectable bear activity on camera traps (0.4 bears per camera night, see Table 1). However, to determine whether scat data corroborated the results of camera trap data, it was necessary to treat these two data sets separately, and Fundy Creek was therefore included as “bear-absent” in the scat analyses. Our response variable for these analyses consisted of the number of mesocarnivore scats encountered on each of two 500-m transect sections at a site, each of which was sampled on two occasions (i.e., n = 4 scat estimates per site, see above). These data were analyzed with Generalized Linear Mixed Effects Models (GLMM) with a Poisson distribution, including site as a random effect. We included as explanatory variables bear presence or absence (based on scats), observer ID (i.e., the researcher who performed the transect section), and sampling occasion (i.e., whether data were from the first or second sampling occasion of a given transect section), as well as the two-way interactions between bear presence or absence and both observer ID and sampling occasion (S1 Table). We used separate GLMMs to test for the effects of bear presence or absence on the relative abundance of (1) all mesocarnivores, (2) just raccoons, and (3) just mink. None of these models showed evidence of overdispersion (ratio of residual deviance to residual DF < 1.3 for all models). The significance of main effects and interactions was tested using Type II Wald’s χ^2^-Test [51].

Finally, our camera traps recorded the time of day at which all animal recordings were made, and we used time data from independent recordings of large carnivores and mesocarnivores to assess whether large carnivores may restrict mesocarnivore temporal activity along shorelines in Clayoquot. Following Ridout and Linkie [52] (see also [53]) we estimated the *coefficient of overlap* (Δ_4_) in temporal activity of mesocarnivores and large carnivores. This metric is based on the kernel density of observations of two animal species/groups across a 24-hr period (ignoring the date on which a given observation was made) and ranges between 0 (no overlap) and 1 (complete overlap) [52,53]. Below we present the estimated temporal overlap between large carnivores and mesocarnivores (Δ_4_) along with the 95% confidence interval based on 10,000 bootstrap replicates (following [53]).

#### Marine prey abundance and intertidal carnivore activity

To examine the relationship between marine prey availability and the intertidal carnivore community, we calculated metrics to describe (1) the overall availability of marine prey, as estimated from quadrat sampling and crab trapping, and (2) the relative abundance of all intertidal foraging mammalian carnivores, as estimated from camera trapping. To calculate overall prey availability at a given site, it was necessary to standardize estimates of the intertidal abundance of shore crabs and prickleback fish, and the shallow subtidal abundance of red rock crabs, as these abundances were estimated using different sampling methodologies. For each prey type, we therefore took the total number of individuals encountered in all quadrats (shore crabs and prickleback fish) or crab traps (red rock crabs) at a given site, and divided this total by the standard deviation of all site-level total abundances for that prey type across the ten sites (i.e., scaled the site-level values by their standard deviation [54]). These standardized prey abundance values for each prey type were then summed to produce our metric of prey availability.

In estimating the relative abundance of all intertidal foraging carnivores at a given site, we focused on those species directly observed to spend large amounts of time foraging in the intertidal; namely, bears, raccoons, and mink. Wolves and cougars both occur and likely hunt in the intertidal (pinnipeds are a major prey item for both wolves and cougars in Clayoquot; [47]), and wolves are known to occasionally forage on small intertidal invertebrates and fish [9]. However direct observations and camera trap data both indicate that, at least at our study sites, the vast majority of intertidal carnivore activity is accounted for by bears and mesocarnivores, and these were the only species observed (either directly or via camera trap videos) actively foraging on small intertidal prey. To estimate site-level intertidal carnivore activity, we used data on the relative abundances of bears and mesocarnivores (raccoons and mink) from camera traps (i.e., individuals per camera-day). Rather than simply summing the total number of intertidal carnivores recorded, it was again necessary to standardize our estimates of bears and mesocarnivores per camera-day at a given site by their respective standard deviations [54] to account for potential differences in the ability of camera traps to detect bears and mesocarnivores, given their substantial differences in body size (bears are several times larger than both raccoons and mink). Standardized values of bears and mesocarnivores per camera-day were then summed to give our intertidal carnivore activity metric. We used linear regression to test for an effect of prey availability on intertidal carnivore activity and, as for the camera trap data described above, conducted our analyses both including (n = 10) and excluding (n = 9) Grice Bay, our only study site at which wolf activity was high. These analyses again produced consistent results in terms of the significance of the relationship between prey availability and intertidal carnivore activity and, for reasons discussed above, we again only present the analyses from which Grice Bay was excluded.

The above intertidal carnivore activity metric provides an estimate of the overall carnivore activity at a site, but does not provide information on the structure of the carnivore community (e.g., whether a site is dominated by bears or mesocarnivores). To quantify intertidal carnivore community structure, we conducted Principal Components Analysis (PCA) on the relative abundances (individuals per camera-day) of bears, raccoons, and mink at each site. Principal components were calculated using the correlation matrix of these three variables, to account for substantially higher variance in bear data than in mesopredator data [55]. The first principal component (PC1) explained 67% of variation among these three carnivore abundance variables. Bear relative abundance was positively associated with PC1 (loading = 0.583), and both raccoon and mink relative abundances were negatively associated with PC1 (loadings = -0.592 and -0.556, respectively), indicating that high positive or negative values of PC1 describe a bear-dominated or mesocarnivore-dominated intertidal carnivore community, respectively (S1 Fig). To provide insight on the relationship between marine prey availability and the structure of the intertidal carnivore community, PC1 scores for each site were used to produce the coloring for points in Fig 2.

### Comparison of intertidal prey abundances where large carnivores are extant or extirpated

We previously reported a strong relationship between intertidal foraging mammalian carnivores and the intertidal prey community in the Gulf Islands, a system from which all native large carnivores (bears, wolves, and cougars) have been extirpated, and where mesocarnivores (predominantly raccoons) are now the only intertidal carnivores [5,36]. Suraci et al. [36] showed that the presence of raccoons on Gulf Islands led to significant reductions in the abundance of marine prey (including shore crabs, prickleback fish, and red rock crabs), relative to Gulf Islands on which raccoons are absent. To assess whether differences in the mammalian carnivore community (i.e., the presence or absence of large carnivores) may lead to differences in the nearshore marine community, we compared intertidal prey abundances in Clayoquot (this study) with abundances of intertidal prey on raccoon-present and raccoon-absent Gulf Islands [36]. As reported in Suraci et al. [36], we used the same methods described above to quantify intertidal prey abundance (quadrat sampling in both the high and mid intertidal zones and shallow subtidal crab trapping) on six raccoon-present and six raccoon-absent Gulf Islands. In the present comparison, for prey species quantified in quadrats we focused our analyses on the intertidal zone in which the species was most abundant (high zone for shore crabs and mid zone for prickleback fish), as this is where Suraci et al. [36] detected the strongest differences between raccoon-present and raccoon-absent Gulf Islands. All quadrat data were expressed as the average number of individuals per m^2^ across all quadrats sampled in either the high or mid intertidal zone at a given site, and red rock crab abundances were expressed as the total number of individuals caught in all crab traps (n = 10 crab traps at each site). We used Wilcoxon’s Rank Sum test with Bonferroni’s correction for multiple comparisons to compare the abundances of (1) medium shore crabs, (2) large shore crabs, (3) prickleback fish, and (4) red rock crabs between Clayoquot (n = 10) and both raccoon-present (n = 6) and raccoon absent (n = 6) Gulf Islands.

Below we report intertidal prey abundances from habitats in which large carnivores are extant (Clayoquot) or extirpated (all Gulf Islands), and where overall carnivore abundance is high (Clayoquot and raccoon-present Gulf Islands) or low (raccoon-absent Gulf Islands). Raccoons are the numerically dominant carnivore in the Gulf Islands, and thus, where raccoons are absent, overall carnivore abundance is indeed relatively low. Suraci et al. [36] describe nocturnal, boat based transects to survey carnivore relative abundance on shorelines in the Gulf Islands, and report that, across all 12 study islands (including raccoon-absent islands), average raccoon relative abundance was 1.77 individuals per km of shoreline surveyed. Two other species of mesocarnivore–mink and river otters (*Lontra canadensis*)–occur in the Gulf Islands and were quantified on these same boat based transects, and were found to occur at low relative abundances across all Gulf Islands surveyed (mean ± SD = 0.24 ± 0.26 individuals km^-1^). Further, the relative abundance of mink and river otters did not differ between raccoon-present and raccoon-absent islands (raccoon-present islands: mean ± SD = 0.23 ± 0.22 individuals km^-1^; raccoon-absent: 0.24 ± 0.31 individuals km^-1^; Wilcoxon’s *W* = 20.5, *P* = 1.0).

## Results

We recorded 418 independent (i.e., separated by ≥ 60 min) images and videos of mammalian carnivores across the 10 sites and 733 camera-days of the study, and identified a total of 478 carnivore scats across all scat transects. These data showed a strong gradient in both mesocarnivore and large carnivore relative abundance among our study sites. Mesocarnivores (raccoons and mink) were present at all sites, as determined by both camera traps and scat transects, but their relative abundance varied substantially across sites, ranging from 0.01 to 0.42 individuals per camera-day and 1.3 to 21.8 scats per 500 m (Table 1). Large carnivores (bears, wolves, and cougars) were detected via camera traps at all but one site (large carnivore scats were found at eight of ten sites), with relative abundances again showing substantial variation between sites (camera trap range = 0 to 1.37 individuals per camera-day, scat transect range = 0 to 14.0 scats per 500 m; Table 1). As noted above, bears were the numerically dominant large carnivore at all but one site (Grice Bay, where wolf activity was exceptionally high, see Table 1).

### Effect of large carnivores on mesocarnivore intertidal habitat use

**Fig 1:**
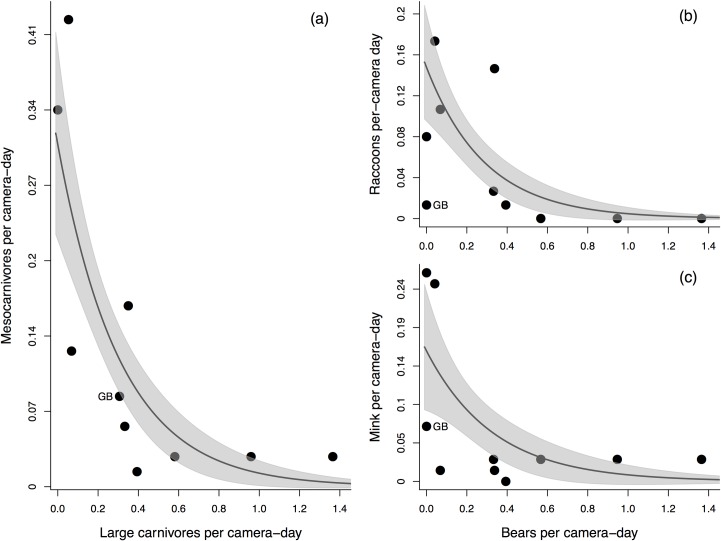
Relationship between large carnivore relative abundance and mesocarnivore relative abundance across ten study sites in Clayoquot, as determined by camera traps. Plots show the relative abundances (i.e., individuals per camera-day) of (a) all large carnivores and all mesocarnivores, (b) bears and raccoons, and (c) bears and mink. Lines represent Poisson GLM estimates of the relationship between mesocarnivore and large carnivore relative abundance, and shaded areas denote standard errors of model estimates. Grice Bay (GB) is indicated in all plots. Grice Bay showed exceptionally high levels of wolf activity and was excluded from the GLM analyses performed on data presented in (b) and (c) (see Methods for details).

The presence and relative abundance of large carnivores significantly affected mesocarnivore intertidal habitat use. The number of mesocarnivores per camera-day at a site decreased significantly with both the total number of large carnivores per camera-day (GLM; *F*_*1*,*8*_ = 14.97, *P* = 0.005; Fig 1A), and the number of bears per camera-day (*F*_*1*,*7*_ = 14.73, *P* = 0.006). When considering mesocarnivore species separately, the number of bears per camera-day also showed a significant negative effect on raccoons per camera-day (*F*_*1*,*7*_ = 9.68, *P* = 0.017, Fig 1B) and a marginally significant negative effect on mink per camera-day (*F*_*1*,*7*_ = 5.12, *P* = 0.058, Fig 1C). Scat transect data corroborated the strong negative effect of bears on mesocarnivore intertidal habitat use, with the number of total mesocarnivore, raccoon, and mink scats per 500 m all being significantly lower at sites where bear scat was detected, relative to sites where no bear scat was found (GLMM; total mesocarnivore scats: Wald’s χ^2^_1_ = 18.99, *P* < 0.001; raccoon scats: χ^2^_1_ = 8.63, *P* = 0.003; mink scats: χ^2^_1_ = 17.57, *P* < 0.001; see S1 Table and S2 Fig).

### Large carnivore and mesocarnivore temporal activity

Mesocarnivores were largely nocturnal, with 81.9% of all mesocarnivore recordings on camera traps (90.5% of raccoon and 75.0% of mink) occurring between the hours of 22:00 and 05:00 (S3 Fig). Overall, large carnivore activity along shorelines was mostly diurnal (96.6% of large carnivore recordings occurred between 05:00 and 22:00; S3 Fig), and bears were almost strictly diurnal in their intertidal foraging behaviour, with 98.7% of bear recordings occurring during daylight hours. Wolf activity was also largely diurnal (80%), while cougar recordings, though entirely nocturnal, were relatively rare (Table 1). The contrasting shoreline activity schedules of mesocarnivores and large carnivores resulted in limited temporal overlap between these groups (*coefficient of overlap* Δ_4_ = 27.6 [95% CI: 19.3–36.2]).

### Relationship between marine prey availability and intertidal carnivore activity

**Fig 2:**
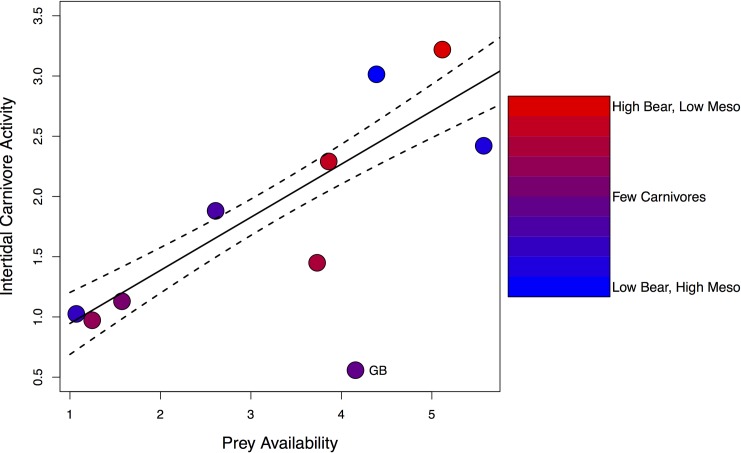
Relationship between intertidal prey availability and the overall activity of intertidal foraging carnivores (bears and mesocarnivores) in Clayoquot. Prey availability and intertidal carnivore activity are scaled metrics of prey abundance and carnivore relative abundance, respectively, and thus do not have units (see Methods for details on calculating these metrics). Coloring of points reflects site-level scores for the first principal component of a PCA describing carnivore community structure, where high values (red) represent bear dominated sites, low values (blue) represent mesocarnivore dominated sites, and middling values (mid-spectrum colors) represent sites with mixed, but relatively low carnivore abundance (see also S1 Fig). Solid and dashed lines represent linear regression estimates (± SE) of the relationship between prey availability and intertidal carnivore activity. Grice Bay (GB) was excluded from this regression analysis, as described in the methods.

Independent of carnivore community composition, marine prey availability at a site was a strong predictor of the overall relative abundance of intertidal foraging carnivores. Overall intertidal carnivore activity increased significantly with marine prey availability (linear regression; *F*_*1*,*7*_ = 22.17, *P* = 0.002, *R*^*2*^ = 0.73; Fig 2), regardless of whether the carnivore community at a given site was dominated by bears or mesocarnivores. The intertidal carnivore PCA (see coloring of points in Fig 2) indicated that sites with high marine prey availability showed high levels of intertidal activity by either bears (red symbols in Fig 2) or mesocarnivores (blue symbols in Fig 2), while both bears and mesocarnivores appeared to avoid sites at which marine prey were relatively scarce (mid-spectrum symbols in Fig 2).

### Intertidal prey abundances where large carnivores are extant or extirpated

**Fig 3:**
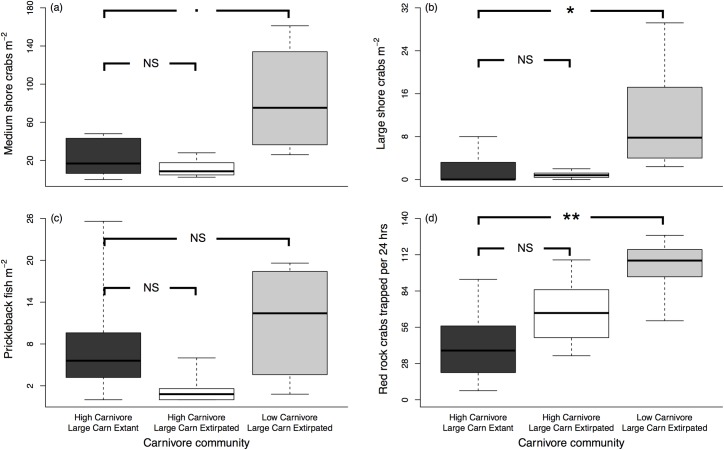
Comparison of intertidal prey abundances where large carnivores are extant (Clayoquot) or extirpated (Gulf Islands). Shown are abundances of (a) medium shore crabs, (b) large shore crabs, and (c) prickleback fish per m^2^; and (d) red rock crabs trapped per 24 hrs from locations where carnivore abundance is high and large carnivores are extant (Clayoquot sound [n = 10 sites], dark boxes), where carnivore abundance is high and large carnivores are extirpated (raccoon-present Gulf Islands [n = 6], white boxes), and where carnivore abundance is low and large carnivores are extirpated (raccoon-absent Gulf Islands [n = 6], grey boxes). Data are presented as standard boxplots; the bold horizontal black lines indicate median values, the box edges represent the 25 and 75% quartiles, and the whiskers signify the range. The endpoints of brackets across the top indicate groups of data compared in statistical tests. N.S., non-significant; 0.06 > *P* > 0.05; * *P* < 0.01; ** *P* < 0.001.

In general, intertidal prey abundances were lower where the relative abundance of carnivores was high (Clayoquot and raccoon-present Gulf Islands) than where carnivore relative abundance was low (raccoon-absent Gulf Islands), consistent with carnivores functioning as top intertidal predators; and there were few differences in intertidal prey abundances between where large carnivores remained extant (Clayoquot) and where they have been extirpated (raccoon-present Gulf Islands), consistent with large carnivores and mesocarnivores having functionally redundant impacts on intertidal prey. Abundances of both medium and large shore crabs (Fig 3A and 3B) were indistinguishable between locations with high carnivore abundance (Clayoquot and raccoon-present Gulf Islands), regardless of carnivore identity, and were marginally (medium shore crabs) or significantly (large shore crabs) lower where large carnivores remained extant (Clayoquot) than where carnivore abundance was low (raccoon-absent Gulf Islands [medium shore crabs: Clayoquot vs. raccoon-present Gulf Islands, Wilcoxon’s *W* = 20.5, Bonferroni-corrected *P* = 0.647; Clayoquot vs. raccoon-absent Gulf Islands, *W* = 50, *P* = 0.057; large shore crabs: Clayoquot vs. raccoon-present, *W* = 38.5, *P* = 0.696; Clayoquot vs. raccoon-absent, *W* = 54, *P* = 0.007]). Similarly, red rock crab abundances (Fig 3D) were not significantly different between locations with high carnivore abundance (Clayoquot and raccoon-present Gulf Islands), while being significantly lower where large carnivores remained extant (Clayoquot) than where carnivore abundance was low (raccoon-absent Gulf Islands [Clayoquot vs. raccoon-present Gulf Islands: *W* = 46, *P =* 0.176; Clayoquot vs. raccoon-absent Gulf Islands: *W* = 59, *P =* 0.001]). Prickleback fish were the exception to this trend (Fig 3C); prickleback fish abundances in Clayoquot were intermediate between those of raccoon-present and raccoon-absent Gulf Islands, and not significantly different from either (Clayoquot vs. raccoon-present Gulf Islands: *W* = 13, *P =* 0.131; Clayoquot vs. raccoon-absent Gulf Islands: *W* = 38, *P =* 0.822).

## Discussion

We found that the composition of the mammalian carnivore community in coastal habitats of Clayoquot was determined both by interactions between carnivore species and by the availability of intertidal prey, indicating that intertidal prey abundance shapes habitat use and competition among this guild of terrestrial predators. Further, carnivore community composition (i.e., the relative abundance of large carnivores and mesocarnivores) appears to have little relevance to impacts on intertidal prey abundance, both within Clayoquot, and when comparing between locations where large carnivores are extant (Clayoquot) or have been extirpated (Gulf Islands), suggesting that foraging by large carnivores and mesocarnivores may have largely similar top-down effects on the intertidal community.

Regardless of community composition, the overall activity of carnivores on shorelines in Clayoquot was strongly related to the availability of intertidal prey (Fig 2). The highest relative abundances of both bears and mesocarnivores (raccoons and mink) were observed at sites with relatively high intertidal prey availability, confirming that this marine food source is an important resource for coastal carnivore populations. Intertidal invertebrates and fish may be a particularly key resource for bears in this area, with individuals being present daily at some of the sites with the highest prey availability (Table 1). Other marine food sources, including herring eggs [37] and spawning salmon [35,56] provide crucial but ephemeral sources of energy and protein for coastal bear populations, and intertidal prey may be important in supporting these populations where and when other marine foods are not available. Indeed, the impact of bears on intertidal prey may shift seasonally with changes in the availability of alternative food sources, though this possibility has not yet been explored.

Raccoons and mink in Clayoquot also appear to track intertidal prey availability, but given the abundance of large carnivores here, access to this resource may be secondary to the presence of potential predators and dominant competitors in driving mesocarnivore activity and habitat use. Mesocarnivore relative abundance was highest at sites where both intertidal prey availability was high and large carnivore relative abundance was low (Fig 2), but declined quickly with increasing large carnivore relative abundance (Fig 1), indicating that large carnivores, and specifically bears, readily exclude raccoons and mink from high quality coastal habitats. Large carnivores may also restrict mesocarnivore temporal activity at coastal sites, further limiting their access to marine resources and potentially reducing the per capita impacts of mesocarnivores on intertidal prey. Raccoon and mink activity was largely nocturnal (S3 Fig), and showed little overlap with the predominantly diurnal shoreline activity of bears and wolves, which may reduce the risk of harassment and predation from these species [41,57,58], but may also prevent these mesocarnivores from exploiting daytime low tides (which mesocarnivores are known to do where large carnivores are absent [5,36]; see below). Studies from continental systems have similarly found that large carnivore activity restricts mesocarnivore local abundance [3,4,59,60] and activity patterns [41], typically through interference competition or direct predation. In our system raccoons and mink may be additionally subjected to exploitative competition with bears, a dominant competitor for intertidal resources, which may further reduce mesocarnivore activity at bear dominated sites through reduced availability of prey, though our data do not allow us to test this hypothesis directly.

Consistent with large carnivores and mesocarnivores having redundant impacts on intertidal prey, the composition of the carnivore community at a given site at Clayoquot did not appear to affect intertidal prey availability (Fig 2)–sites with the highest levels of either bear or mesocarnivore (raccoon and mink) activity all showed a similarly high availability of intertidal prey. This could indicate that carnivore foraging has limited effects on the abundance of intertidal species, however this is unlikely, given our previous research experimentally demonstrating that raccoon foraging significantly reduces the abundances of the same suite of intertidal prey [5,36]. Instead, we suggest that the similarity in prey availability between sites with high bear activity and sites with high raccoon and mink activity indicate a similarity between large carnivores and mesocarnivores in their top-down effects on the intertidal community. This is further supported by our comparison of intertidal prey abundance between Clayoquot, where bears are the dominant intertidal foragers, and the Gulf Islands, where bears and all other large carnivores are absent, and mesocarnivores (mainly raccoons) are now the only mammalian predators of intertidal prey. This comparison (Fig 3) shows that, for most prey types, abundances are similar between locations with high carnivore abundance (Clayoquot and raccoon-present Gulf Islands), regardless of whether large carnivores (specifically bears) are extant (Clayoquot) or extirpated (raccoon-present Gulf Islands), and are substantially lower than where overall carnivore abundance is low (raccoon-absent Gulf Islands).

Despite the similarities in habitat type (cobble-boulder beaches) and wave exposure (sheltered water ways, see above) between our study sites in Clayoquot and the Gulf Islands, the geographic separation between these locations (approximately 140 km) may affect the baseline abundances of intertidal prey (e.g., through potential differences in nutrient availability). Nonetheless, the broad overlap in intertidal prey abundance between Clayoquot and raccoon-present Gulf Islands points to possible functional redundancy between large carnivores and mesocarnivores, and specifically between bears and raccoons, in their role as top predators in intertidal communities. In the context of our study system, redundancy would mean that the top-down effects of mammalian carnivores on intertidal prey are equivalent regardless of whether the intertidal carnivore community contains just raccoons or both raccoons and bears, and could occur if raccoons increase in abundance and/or per capita impacts on intertidal prey [22–24] following large carnivore extirpation. Our previous research suggests that raccoon relative abundance along shorelines is substantially higher in the Gulf Islands than in Clayoquot [36], and that the absence of native large carnivores in the Gulf Islands allows raccoons to dramatically increase their impacts on intertidal prey, in part by allowing raccoons to exploit both nocturnal and diurnal low tides [5,36]. In Clayoquot, raccoon impacts are likely substantially lower, as their presence in the intertidal is evidently strongly restricted by large carnivore activity. However, given the high degree of omnivory and dietary overlap between bears and raccoons, this does not translate to higher abundances of intertidal prey. Instead, predation by bears appears to suppress intertidal prey to similarly low levels.

Further research is needed to confirm the observed similarity between bear and raccoon impacts on the intertidal prey community. However if this pattern holds true, it suggests that, when released from top down control by, and competition with, large carnivores, mesocarnivores may in some cases fill the now vacant niche of their former predator/competitor. This is an unexpected potential outcome of mesopredator release, studies of which often detect substantial differences in the prey community in the presence and absence of large carnivores (e.g., [3,4]) rather than the broad similarities observed here. The potential for mesocarnivores to partially assume the functional role of an extirpated large carnivore may exist more generally in carnivore communities containing highly omnivorous species, and could provide an important source of resiliency against the disturbance caused by anthropogenic large carnivore declines [24]. Omnivory has been suggested to be a stabilizing force in food webs in general [25,61], but the potential for omnivory among carnivores to act as a buffer against anthropogenic species loss has not been explored.

It is important to note, however, that bears and raccoons are both generalist foragers whose ecological effects are likely to extend far beyond the intertidal community [35,62,63]. For instance, raccoons are major predators on nesting songbirds, both in the Gulf Islands and elsewhere, and are frequently implicated in songbird declines [36,62,64], while bears are a crucial component of the well-studied salmon-carnivore system described above, capable of removing large numbers of salmon from spawning streams [31,35]. Thus the functional overlap between these two species may be limited to their effects on the intertidal prey community, and there is no question of raccoons fully replacing the ecological role of bears in areas where they have been extirpated.

The shoreline is an important source of food for many coastal carnivore populations [27–29], and here we show that the community of invertebrates and small fish in rocky intertidal habitats provide a valuable resource for these terrestrial predators, helping to shape habitat use and competition between carnivore species. Coastal areas across the globe also support high-density human populations [65], and the composition of the mammalian carnivore community in many regions may largely be a product of anthropogenic disturbance, dominated by human-tolerant mesocarnivores and lacking large carnivores. Our results raise the possibility that, in some cases, a shift from an intact carnivore community to one with no large carnivores may have limited consequences for the intertidal community due to redundancy in the functional role of mammalian carnivores as intertidal predators. However, the otherwise well-described repercussions of worldwide large carnivore declines [66] have not been well assessed for coastal ecosystems, and further research is necessary to understand the impacts of large carnivore losses on intertidal communities.

## Supporting Information

S1 Dataset Supplementary Data. This file includes all data required to perform the statistical analyses presented in this paper.
(XLSX)

S1 Fig Biplot of the first two Principal Components from a PCA of the relative abundances (individuals per camera-day) of bears, raccoons, and mink at each study site. Factor loadings for the three carnivore species are shown in red. Names of the 10 study sites, shown in black, correspond to those presented in Table 1. PC1 describes the relative abundance of mesocarnivores (raccoons and mink) and large carnivores (bears; see text for details), and PC1 scores for each site were used to produce the coloring for points in Fig 2.
(PDF)

S2 Fig Effect of bear presence or absence (as determined by scats) on the number of mesocarnivore scats detected. Number of (a) total mesocarnivore, (b) raccoon, and (c) mink scats detected at sites in Clayoquot Sound at which bear scat was either present or absent. Values are the mean (± SE) number of scats detected per 500-m transect section walked at each site.
(PDF)

S3 Fig Temporal activity of large carnivores and mesocarnivores across all sites in Clayoquot Sound. Bars are the total number of independent mesocarnivore (white bars) and large carnivore (black bars) recordings on camera traps made during each one-hour period across the 24-hour day. Dashed lines represent the approximate beginning and end of the diurnal period during the mid-to-late summer in Clayoquot, when this study was conducted.
(PDF)

S1 Table Scat data model results. Full results from Poisson Generalized Linear Mixed Effects Models testing the effects of bear scat presence or absence, observer, and sampling occasion on the number of mesocarnivore scats detected at a site. P-values shown in bold are significant at α = 0.05.
(PDF)
