# Supplementary figures and images for: Do Large Carnivores and Mesocarnivores Have Redundant Impacts on Intertidal Prey?
Source: PLoS One. 2017 Jan 13;12(1):e0170255. doi: 10.1371/journal.pone.0170255 (PMC5235380; doi:10.1371/journal.pone.0170255)

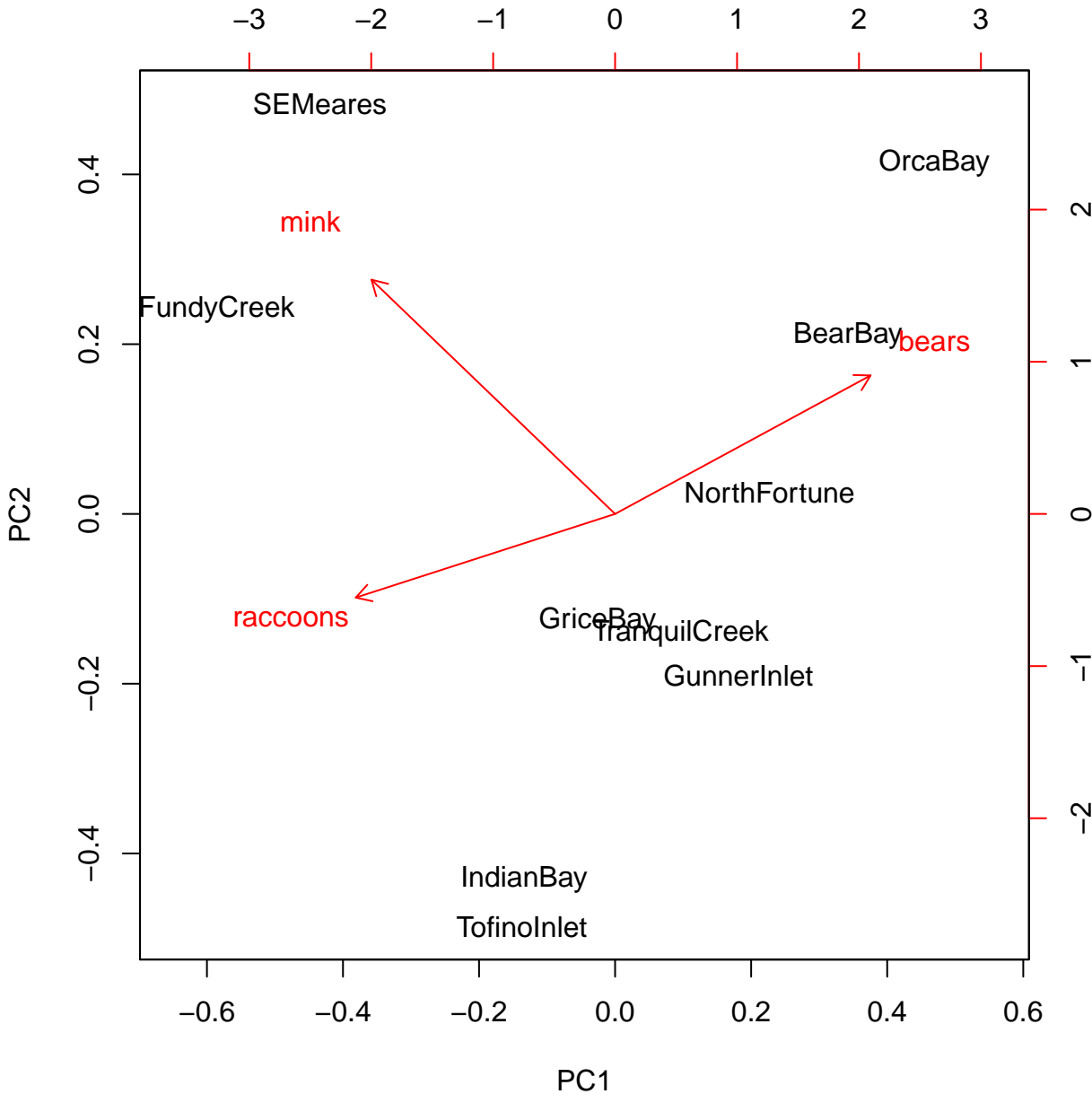

Supplement: S1 Fig — Factor loadings for the three carnivore species are shown in red. Names of the 10 study sites, shown in black, correspond to those presented in Table 1. PC1 describes the relative abundance of mesocarnivores (raccoons and mink) and large carnivores (bears; see text for details), and PC1 scores for each site were used to produce the coloring for points in Fig 2. (PDF) [file pone.0170255.s002.pdf]

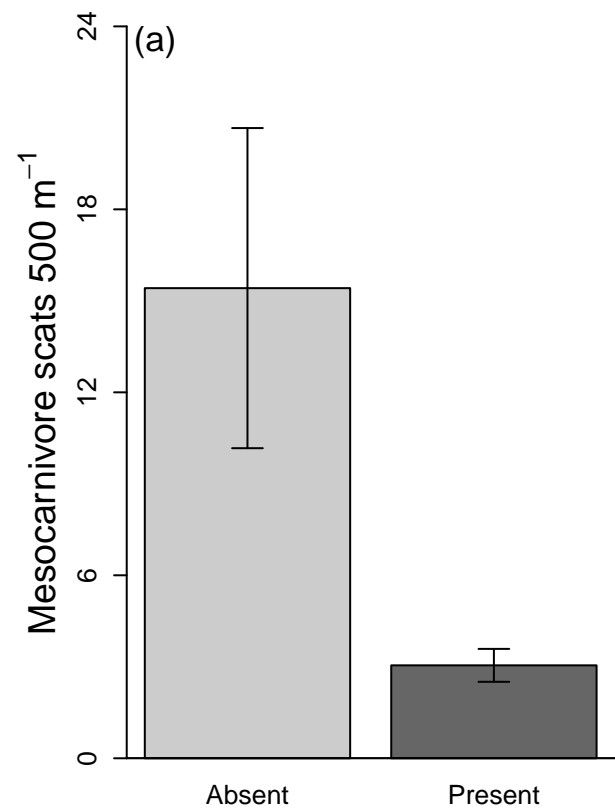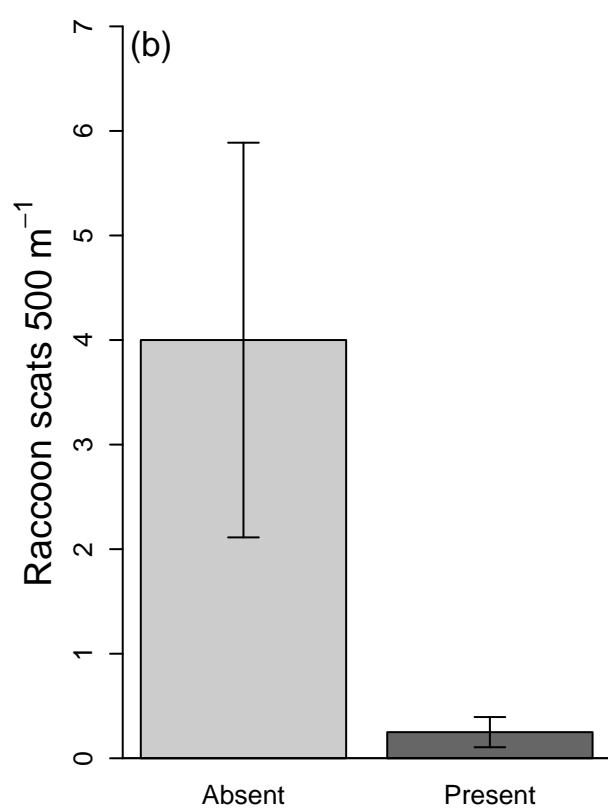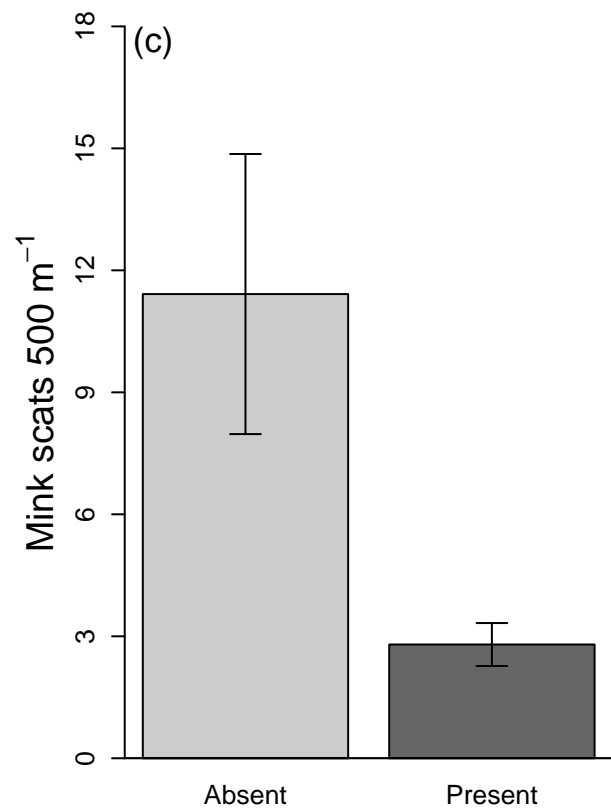

Bear scat present or absent at site

Supplement: S2 Fig — Number of (a) total mesocarnivore, (b) raccoon, and (c) mink scats detected at sites in Clayoquot Sound at which bear scat was either present or absent. Values are the mean (± SE) number of scats detected per 500-m transect section walked at each site. (PDF) [file pone.0170255.s003.pdf]

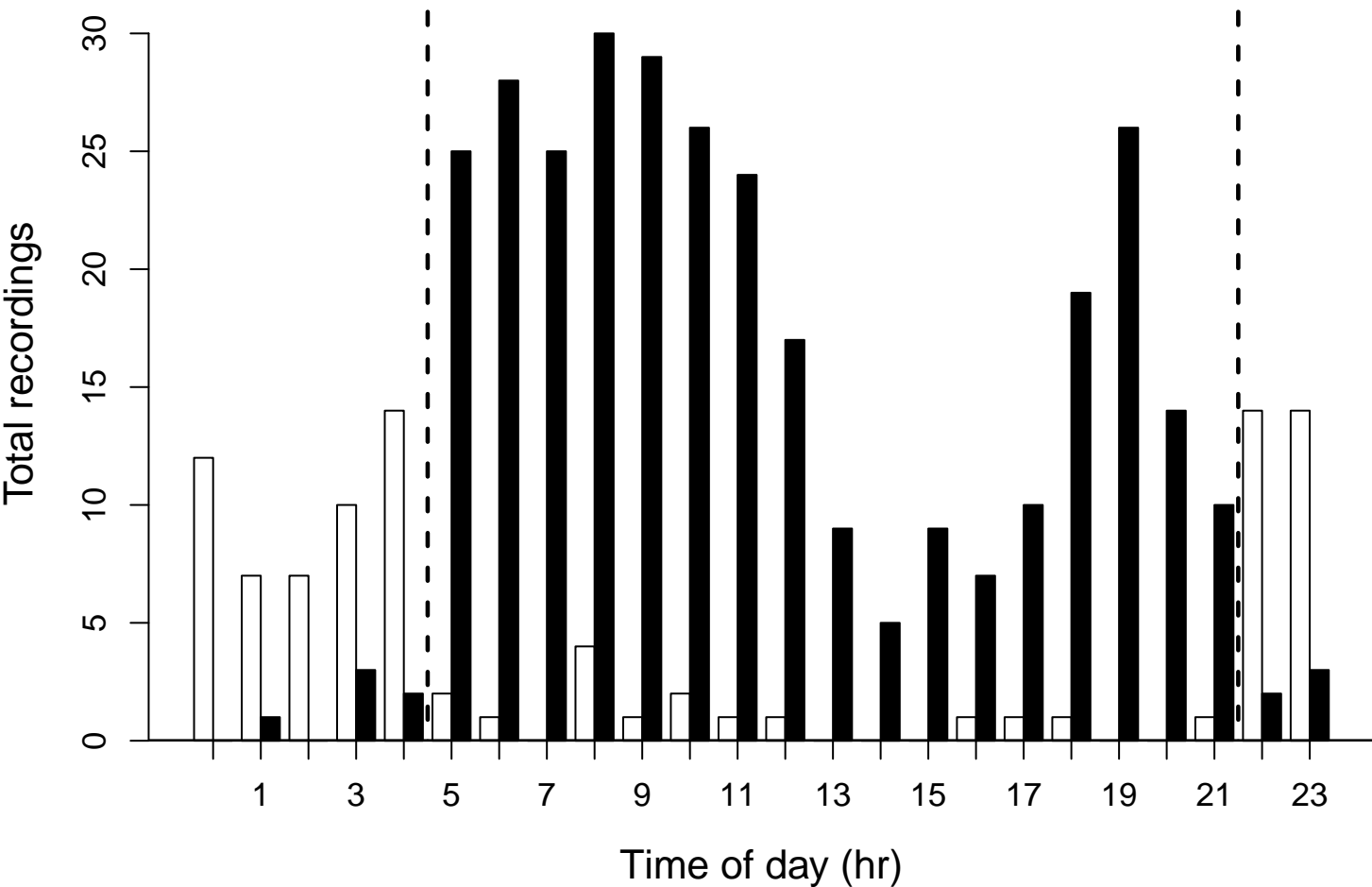

Supplement: S3 Fig — Bars are the total number of independent mesocarnivore (white bars) and large carnivore (black bars) recordings on camera traps made during each one-hour period across the 24-hour day. Dashed lines represent the approximate beginning and end of the diurnal period during the mid-to-late summer in Clayoquot, when this study was conducted. (PDF) [file pone.0170255.s004.pdf]
