# Supplementary material for: Do Large Carnivores and Mesocarnivores Have Redundant Impacts on Intertidal Prey?
Source: PLoS One. 2017 Jan 13;12(1):e0170255. doi: 10.1371/journal.pone.0170255 (PMC5235380; doi:10.1371/journal.pone.0170255)
Supplement: S1 Table — Full results from Poisson Generalized Linear Mixed Effects Models testing the effects of bear scat presence or absence, observer, and sampling occasion on the number of mesocarnivore scats detected at a site. P-values shown in bold are significant at α = 0.05. (PDF) [file pone.0170255.s005.pdf]

Table S1. Full results from Poisson Generalized Linear Mixed Effects Models testing the effects of bear scat presence or absence, observer, and sampling occasion on the number of mesocarnivore scats detected at a site. P-values shown in bold are significant at α = 0.05.

	All Mesocarnivores			Raccoons			Mink		
Model Term	Wald's χ^2	DF	P-value	Wald's χ^2	DF	P-value	Wald's χ^2	DF	P-value
Bear Scat Presence/Absence	18.99	1	**< 0.001**	8.63	1	**0.003**	17.56	1	**< 0.001**
Observer	5.24	1	**0.022**	16.06	1	**< 0.001**	0.15	1	0.701
Sampling Occasion	26.96	1	**< 0.001**	2.17	1	0.141	25.43	1	**< 0.001**
Bear Scat x Observer	0.15	1	0.696	0.03	1	0.854	1.54	1	0.214
Bear Scat x Sampling Occasion	1.05	1	0.306	1.14	1	0.287	0.28	1	0.595
